# Preoperative Assessment for Event-Free Survival With Hepatoblastoma in Pediatric Patients by Developing a CT-Based Radiomics Model

**DOI:** 10.3389/fonc.2021.644994

**Published:** 2021-04-16

**Authors:** Yi Jiang, Jingjing Sun, Yuwei Xia, Yan Cheng, Linjun Xie, Xia Guo, Yingkun Guo

**Affiliations:** ^1^West China Second University Hospital, Sichuan University, Chengdu, China; ^2^Huiying Medical Technology, Beijing, China

**Keywords:** pediatric, hepatoblastoma, computer tomography imaging, prognosis, radiomics, nomogram

## Abstract

**Objective:** To explore a CT-based radiomics model for preoperative prediction of event-free survival (EFS) in patients with hepatoblastoma and to compare its performance with that of a clinicopathologic model.

**Patients and Methods:** Eighty-eight patients with histologically confirmed hepatoblastoma (mean age: 2.28 ± 2.72 years) were recruited from two institutions between 2002 and 2019 for this retrospective study. They were divided into a training cohort (65 patients from institution A) and a validation cohort (23 patients from institution B). Radiomics features were extracted manually from pretreatment CT images in the portal venous (PV) phase. The least absolute shrinkage and selection operator (LASSO) Cox regression model was applied to construct a “radiomics signature” and radiomics score (Rad-score) for EFS prediction. Then, a nomogram incorporating the Rad-score, updated staging system, and significant variables of clinicopathologic risk (age, alpha-fetoprotein (AFP) level, histology subtype, tumor diameter) as the radiomic model, clinicopathologic model, and combined clinicopathologic-radiomic model were built for EFS estimation in the training cohort, the performance of which was assessed in an external-validation cohort with respect to clinical usefulness, discrimination, and calibration.

**Results:** Nine survival-relevant features were selected for a radiomics signature and Rad-score building. Multivariable analysis revealed that histology subtype (*P* = 0.01), PV (*P* = 0.001) invasion, and metastasis (*P* = 0.047) were independent risk factors of EFS. Patients were divided into low- and high-risk groups based on the Rad-score with a cutoff of 0.08 according to survival outcome. The radiomics signature-incorporated nomogram showed good performance (*P* < 0.001) for EFS estimation (C-Index: 0.810; 95% CI: 0.738–0.882), which was comparable with that of the clinicopathological model for EFS estimation (C-Index: 0.81 vs. 0.85). The radiomics-based nomogram failed to show incremental prognostic value compared with that using the clinicopathologic model. The combined model (radiomics signature plus clinicopathologic parameters) showed significant improvement in the discriminatory accuracy, along with good calibration and greater net clinical benefit, of EFS (C-Index: 0.88; 95% CI: 0.829–0.933).

**Conclusion:** The radiomics signature can be used as a prognostic indicator for EFS in patients with hepatoblastoma. A combination of the radiomics signature and clinicopathologic risk factors showed better performance in terms of EFS prediction in patients with hepatoblastoma, which enabled precise clinical decision-making.

## Introduction

Hepatoblastoma (HB) is the primary hepatic malignancy that occurs in childhood worldwide. With the prevalence of HB being on the rise, an annual incidence of 1.5 cases/million people has been documented (1, 2). Complete resection of the liver is the first-line treatment for early-stage HB with localized lesions (3–5). However, a considerable proportion of patients with unresectable advanced-stage HB requires preoperative chemotherapy (3). The survival outcomes of pediatric patients with HB have improved substantially over recent decades, primarily due to developments in surgical methods and therapy intensification (3, 6). Nevertheless, the optimal combination strategy of chemotherapy and surgery and the increased risk of toxicities from cumulative chemotherapy have not been addressed (7, 8). Thus, therapy should be based on the identification of patients at high risk of a poor outcome in HB.

In recent decades, several prognostic and risk factors of HB have been reported (9–13). Several prospective studies have been conducted by four major research teams: the Children's Oncology Group (COG); the International Childhood Liver Tumors Strategy Group (SIOPEL); the Japanese Study Group for Pediatric Liver Tumors (JPLT); and the German Society for Pediatric Oncology and Hematology (GPOH). These teams have developed risk-stratified stratagems to improve survival outcomes (14). One remarkable achievement was the Pretreatment Extension of Disease (PRETEXT) system introduced by SIOPEL, which is used widely for the preoperative diagnosis of HB based on imaging assessment (15). However, each study has used different risk-stratification strategies, which yielded dissimilar outcome predictions. In 2018, a final, uniform, global hepatoblastoma stratification (HS) system was established by the Children's Hepatic tumors International Collaboration (CHIC) based on pooled trial data from cooperative groups (1,605 patients from JPLT, GPOH, COG, and SIOPEL). The system refined the individual prognostic variables with age and the PRETEXT stage, together with annotation factors, alpha-fetoprotein (AFP) concentration, metastases, and tumor resectability (4). Gradually, patients with HB benefitted from better stratification to select the most appropriate therapeutic option. Notwithstanding this accomplishment, this risk-stratification model was neither finalized with regard to the histology type nor adapted to the treatment response, as it was somewhat complex in terms of clinical utility in smaller patient cohorts (4, 10, 11, 16). Meanwhile, preoperative PRETEXT has moderate accuracy with a tendency of over-staging, and its diagnostic accuracy is dependent upon imaging technology/equipment to some extent (4, 17). The creation of a feasible, simple, and practical prognostic stratification model to identify risks in patients with HB remains a major challenge.

Medical imaging is vital in clinical management to aid in decision-making and guide “individualized” treatment (18). Over recent years, rapid advances in “big data” and artificial intelligence have led to breakthroughs in “data mining” during analyses of medical images. These advances have created the field of “radiomics,” which has allowed the prediction of clinical endpoints in various types of cancer (19–23). A nomogram model incorporating radiomics features and clinicopathologic factors seems to improve the prognostic accuracy (24, 25). Taking into consideration the heterogeneity of HB is crucial for risk stratification and the prognosis (16). Also, the limitation of histology samples means that the full information about a histological type within a tumor is lacking, which can compromise management (26). Also, patients with an identical PRETEXT stage can show variations in recurrence and survival outcomes (27). These observations demonstrate that the current staging system for HB does not provide adequate prognostic information about the biological heterogeneity of HB. In this regard, radiomics (i.e., characterizing tumor phenotypes by extracting information about the biological processes of tumors from medical images) could facilitate the prediction of tumor progression.

We have no evidence of obtaining a “radiomics signature” to predict the survival outcome of HB. We developed a predictive model comprising radiomics based on CT-derived images and clinical features to forecast event-free survival (EFS) in patients with HB and to assess its additional value to the staging system.

## Materials and Methods

### Ethical Approval of the Study Protocol

The protocol for this retrospective multicenter study was approved by the Ethics Committee of the West China Second University Hospital within Sichuan University (Sichuan, China). The requirement for written informed consent was waived.

### Inclusion Criteria

The criteria for study inclusion were as follows: (i) age <18 years; (ii) histopathologic diagnosis of HB; (iii) preoperative CT of the abdomen and imaging of sufficient quality for analyses; and (iv) complete medical records with data on the surgical procedure and follow-up.

### Patients

The database of our institution was used to collect medical records from January 2009 to August 2019. The internal training cohort comprised 65 patients with HB confirmed by histology who underwent hepatectomy or needle biopsy at the West China Second University Hospital. The data for the external validation cohort were from 23 consecutive patients from the West China Second University Hospital of Sichuan University between January 2012 and December 2019 with identical enrolment criteria. In total, 88 patients were enrolled (57 males and 31 females; mean age, 2.28 ± 2.72 years; age range, 0.1–12.9 years) in the present study. Clinicopathologic data were recorded for each patient. Surgical excisions or tumor-biopsy samples were reviewed by a very experienced pathologist to assess the histology subtype of the tumor. Histopathology confirmation was done with a surgical specimen in 68 patients and with core-needle biopsy in 20 patients.

Clinical information (sex, age, tumor diameter, AFP concentration, clinical stage, surgical notes, treatment regimen, chemotherapy, survival at the time of final follow-up, CT-reported imaging features) at baseline was documented. Tumor stage and preoperative risk assessment were reclassified according to the CHIC- Hepatoblastoma Stratification (HS) and the 2017 PRETEXT systems (4, 13).

### Follow-Up

Event-free survival was the primary endpoint in this study (28). EFS is defined as the period from the date of CT examination to the date of the first relapse, of development of a second malignancy, of disease progression, of death, or of final follow-up, as appropriate (4). The minimum duration of follow-up for EFS was 10 months. The maximum duration of follow-up was 143 (median, 27) months. CT scan of the chest and abdomen was done every 2 months in the first year, every 3 months in the second year, and every 6 months in the third year after surgery, and was also done annually; this strategy was in line with the follow-up procedure of West China Second University Hospital.

### CT Image Acquisition and Feature Analysis

Contrast-enhanced CT of the abdomen was conducted <7 days before surgery. The data of CT images were obtained using two multidetector-row CT systems (NeuViz 128, Neusoft, Beijing, China; Discovery CT750 HD, GE Healthcare, Piscataway, NJ, USA) at two institutions. The detailed imaging protocol is described in Supplementary Material I. The morphologic characteristics of HB lesions on CT images were documented as CT-reported features for the possible prognostic factors of EFS. These factors were the diameter, location, morphology (round/lobulated vs. irregular), margin (well-circumscribed vs. ill-defined), and density modification (homogeneous vs. heterogeneous) of the tumor, as well as the presence of hemorrhage, cystic/necrotic components and calcifications, capsular retraction, tumor vessels, perihepatic effusion, collateral veins, and vascular invasion. Distant metastasis in remote organs was also recorded. In addition, an imaging-based 2017 PRETEXT staging system and related annotation factors were applied for the grouping of tumor extent and analyses of image features (4). The factors were vascular involvement (V, hepatic vein/inferior vena cava; P, portal vein), multifocality (F), tumor rupture (R), extrahepatic tumor extension (E), the involvement of the caudate lobe (C), lymph-node metastases (N), and distant metastases (M). CT scans of the chest and MRI of the brain were done in all patients to investigate distant metastases before treatment.

### Collection of Clinicopathologic Risk Factors

We collected the clinicopathologic characteristics recognized as being significant risk factors according to the risk stratification for HB set by CHIC-HS (15). That is, the risk stratification of patients was classified into three groups (very low risk/low risk; intermediate risk; high risk), and the age was divided into three groups (≤2, 3–7, and ≥8 years). Groups for the serum AFP concentration (≤100, 101–1,000, and >1,000 ng/ml) were not included in the analyses because the AFP concentration was much more than 100 ng/ml for most patients and only three patients had an AFP concentration in the range of 101–1,000 ng/ml. We combined the very-low-risk group and the low-risk group into one group because of the identical EFS in these two groups. In addition, a combined factor of VPEFR was identified as positive (VPEFR+) if one of the V, P, E, F, or R factors, as described by CHIC-HS, were present (4). The 2014 International Consensus Histology Classification for HB (29) was used for the reallocation of histology subtypes for patients in our study.

### Analyses of Radiomics Features Based on CT

Tumor segmentation and extraction of radiomics features were carried out on the Radcloud platform 3.1.0 (http://radcloud.cn/; Huiying Medical Technology, Beijing, China). CT scans were done at different centers. Hence, the corresponding image preprocessing along with filtering of image data for normalization of CT images was undertaken to obtain more robust radiomics features (Supplementary Material II). To segment the volume of interest (VoI) of tumors for further analyses, two radiologists (JY and CY, with 8 and 12 years of experience in the interpretation of abdominal CT images in children, respectively) delineated the region of interest (RoI) manually (30) along the contour of the lesion in layers on the portal venous (PV)-phase image in a blinded manner. All depicted RoIs were delineated strictly using identical criteria and validated visually by the same expert (JY). The “pyradiomics” package within Python 3.8.1 (https://pyradiomics.readthedocs.io/) was employed to extract 1,409 quantitative imaging features from images of the PV-phase on CT. The detailed information of radiomics features are depicted in Supplementary Material III. All radiomics features were complied with definitions as delineated by the Imaging Biomarker Standardization Initiative (30).

### Feature Selection and Building of a Prediction Model

The least absolute shrinkage and selection operator (LASSO) Cox regression model was adopted to reduce the redundancy of high-dimensional features and to select the most useful prognostic features correlated to EFS. Then, the radiomics score (“Rad-score”) based on a selected radiomics signature was built using a Cox regression model for predicting the risk of disease progression in the training cohort. In addition to the radiomics model, independent prognostic factors were selected gradually by univariate and multivariate Cox regression analyses carried out in the training cohort to form a clinicopathologic model and an incorporated clinicopathologic-Rad-score (CR) model for EFS prediction. Then, a nomogram was created for the visualization of EFS prediction.

### Survival Analysis Based on the Radiomics Signature

The difference in EFS between the Rad-score and clinicopathological risk-factor groups was analyzed using the Kaplan–Meier survival curves and the log-rank test in the training cohort. Then, the result was applied to the validation cohort. Meanwhile, the radiomics signature was used to divide patients of the training cohort into low- and high-risk groups based on the optimal Rad-score cutoff point (which was identified in log-rank statistics), and verification was done in the validation cohort.

### Evaluation of the Model Using a Radiomics Signature

**Figure 1:**
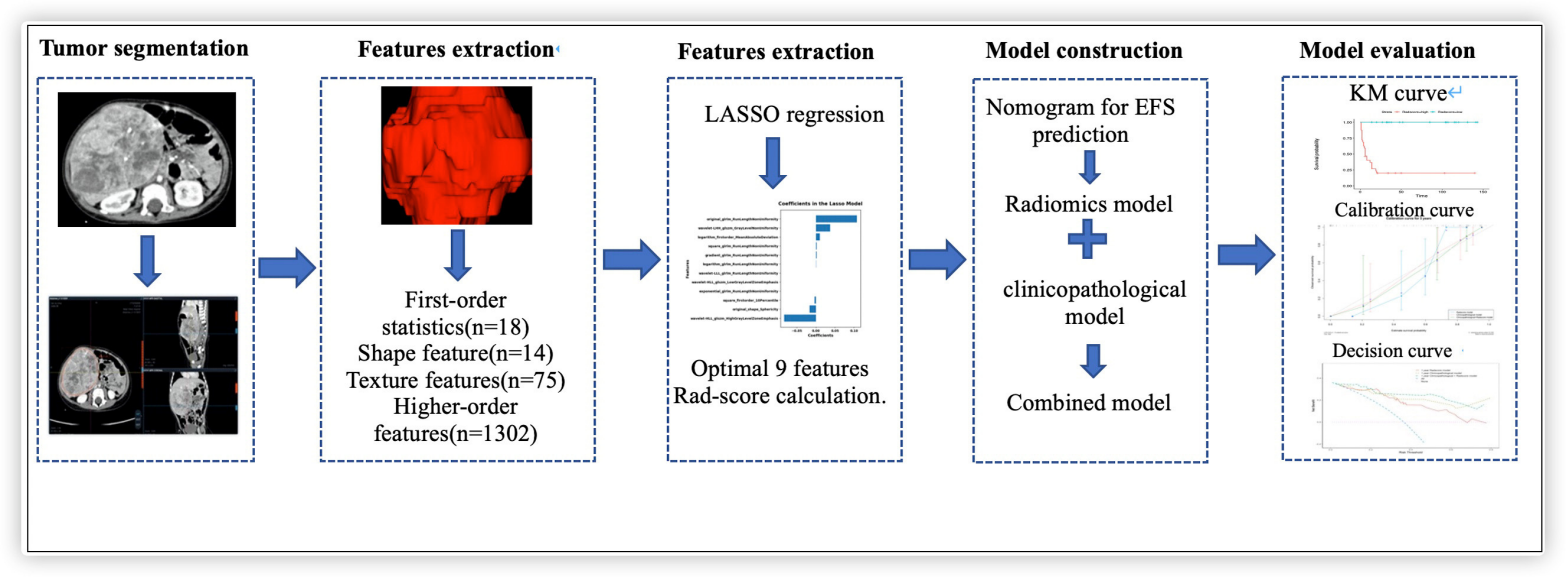
Radiomics framework of predicting the EFS of patients with hepatoblastoma.

The prediction performance of our model was evaluated according to discrimination, calibration, and clinical usefulness. The discrimination performance was quantified using Harrell's Concordance Index (C-Index). A C-Index of ~0.7 indicates a good predictive value. Calibration was explored based on consistency between estimated 3-year survival and actual 3-year survival in the corresponding calibration curves in the training and validation cohorts. Analyses of decision curves denoted clinical usefulness based on the net benefit of the model across different threshold probabilities. The study workflow, comprising of image collection, lesion segmentation, extraction of radiomics features, feature selection, construction of models in the training cohort, and evaluation of the performance of prediction models in the test cohort, is elaborated in Figure 1.

### Statistical Analysis

Normalization of features, selection of features, and model construction were undertaken using Python 3.8.1 (www.python.org/). The “scikit-learn” (https://scikit-learn.org/), “pyradiomics” (https://pyradiomics.readthedocs.io/), and “matplotlib” (https://matplotlib.org/) packages were applied. Other statistical analyses were accomplished with SPSS 26.0 (IBM, Armonk, NY, USA) and R 4.0.3 (www.R-project.org/). *P* < 0.05 was considered significant. EFS was analyzed by the Kaplan–Meier method. The log-rank test was employed to compare the outcome between patients with different risk factors. Characteristics at baseline in the training and validation cohorts were assessed by an independent-sample *t*-test, the two-tailed χ^2^-test, or the Fisher's exact test as appropriate. Univariate and multivariate Cox regression analyses were undertaken to screen for the significant predictors of EFS. Factors with *p* < 0.10 in the univariable analysis were provided as input to the multivariable analysis. The variable risk was denoted as a hazard ratio (HR) with a corresponding 95% confidence interval (CI).

## Results

### Patient Characteristics at Baseline

The characteristics of the patients at baseline in the training and validation cohorts are detailed in Table 1. Of the 88 patients included in this study at final follow-up, the mean duration of follow-up was 42.43 months and the median duration of EFS was 19 months. The shortest duration of EFS was 1 month. The percentage of EFS at 5 years was 73.8% in the training cohort and 78.3% in the validation cohort. Thirty-four events were observed for the EFS calculation: 29 patients had disease progression, 17 patients relapsed, and 16 died after surgery. Most patients (85/88) had a serum AFP of >1,000 ng/ml. Only three patients had a serum AFP level between 100 and 1,000 ng/ml. Of these 88 patients, 14 (16.6%) were diagnosed with distant metastasis upon their first evaluation. Lung metastases were documented in 13 patients, and one patient had mandibular-bone metastases. The histology subtype was available for all patients. There was no significant difference between the two cohorts with respect to clinicopathologic characteristics or survival (*P* = 0.073–0.903).

**Table 1:** Baseline patient and tumor characteristics according to the radiomics score in the training and validation cohorts.

**Characteristic**	**Category**	**Training**	**Validation**	***P-*value**
Number of patients		65	23	
Age at initial diagnosis (years)	Mean age (y)	2.99 ± 2.44	3.09 ± 3.32	0.407
	≤2	53 (81.5%)	13 (56.5%)	
	3–7	8 (12.3%)	8 (34.7%)	
	≥8	4 (6.1%)	2 (8.6%)	
Sex				0.648
	Male	43 (14%)	14 (60.8%)	
	Female	22 (33.8%)	9 (39.1%)	
Serum AFP concentration, ng/mL				0.395
	≤100	0	0	
	101–1,000	2 (3%)	1 (4.3%)	
	>1,000	63 (96.9%)	22 (95.6%)	
Histological subtype				0.903
	Fetal	23 (35.3%)	7 (30.4%)	
	Embryonal	2 (3%)	1 (4.3%)	
	Epithelial mixed	16 (24.6%)	5 (21.7%)	
	Mixed epithelial/mesenchymal	24 (36.9%)	10 (43.4%)	
PRETEXT group				0.196
	I	9 (13.8%)	7 (30.4%)	
	II	31 (47.6%)	9 (39.1%)	
	III	14 (21.5%)	4 (17.3%)	
	IV	11 (16.9%)	3 (13.0%)	
Annotation factors				
V (HV or IVC involvement)	Yes	12 (18.4%)	1 (4.3%)	0.101
P (PV involvement)	Yes	17 (26.1%)	2 (8.6%)	0.08
E (extrahepatic tumor extension)	Yes	2 (3%)	2 (8.6%)	
F (multifocality)	Yes	17 (26.1%)	4 (17.3%)	0.397
R (tumor rupture)	Yes	3 (4.6%)	2 (8.6%)	0.468
C (caudate involvement)	Yes	9 (13.8%)	2 (8.6%)	0.073
N (lymph node metastasis)	Yes	6 (9.2%)	1 (4.3%)	0.457
M (distant metastasis)	Yes	10 (15.3%)	4 (17.3%)	0.662
One or more of V, P, E, F, R	Yes	26 (40%)	6 (26%)	0.233
CHIC-HS risk stratification				0.615
	Very low/low	35 (53.8)	15 (65.2%)	
	Intermediate	13 (20%)	3 (13.0%)	
	High	17 (26.1%)	5 (21.7%)	
Number of deaths		17 (26.1%)	5 (21.7%)	0.674
Follow-up time (month)	Median	30	27	0.391
	Maximum	143	97	
EFS	Median (month)	19	20	0.569
	Event	27 (41.5%)	7 (30%)	0.347
	No event	38 (58.4%)	16 (69.5%)	
Preoperative chemotherapy		13 (20%)	5 (21.7%)	
Surgical resection		46 (70%)	15 (65.2%)	
Orthotopic liver transplantation		1	0	
Resection of pulmonary metastases		1	0	

*AFP, alpha-fetoprotein; PRETEXT, 2017 PRE-Treatment EXTent of tumor; HV, hepatic vein; IVC, inferior vena cava; PV, portal vein; CHIC-HS, Children's Hepatic tumors International Collaboration-Hepatoblastoma Stratification; EFS, event-free survival*.

The inter- and intra-observer reproducibility of extraction of radiomic features were high. Therefore, all outcomes were based on the measurements taken by the first radiologist (JY).

### CT Features

The mean value for the maximum dimension of the tumor was 10.7 cm (range: 4.2–19). The tumors had delineated boundaries (41/88, 46.6%), capsules (79/88, 89.8%), collateral circulation (17/88, 19.3%), bleeding (17/88, 19.3%), and vessels (35/88, 39.8%), and some tumors were heterogeneous (79/88, 89.8%) at presentation. Also, 21/88 (23.9%) tumors were multifocal, whereas 21/88 (23.9%) had perihepatic effusion. Cystic/necrotic components were present in 83/88 (94.362%) tumors, and calcifications were present in 39/88 (44.3%) lesions. The calcifications were punctate, coarse, or speckled. The capsular retraction was noted in 67/88 (76.1%) of tumors.

### Collection of Clinicopathologic Prognostic Factors

The identified significant association factors for EFS were determined by univariable and multivariable logistic regression analysis. We found that CHIC-HS risk stratification; PRETEXT grade; histology subtype; the PRETEXT annotation factors, M and P; and VPERF+ were independent of clinical prognostic risk factors (Table 2). Multivariable Cox proportional hazard analysis showed that the PRETEXT annotation factor P was the strongest predictor (HR, 7.43; 95% CI: 2.134–25.911; *P* = 0.001). The histology subtype showed a similar significance in predicting EFS (HR, 1.364; 95% CI: 1.075–1.731; *P* = 0.010) whereas distant metastasis showed borderline significance for EFS prediction (HR, 0.150; 95% CI: 0.023–0.977; *P* = 0.047).

**Table 2:** Univariate and multivariate analysis of event-free survival for patients in training cohort.

**Variables**	**Univariate**		**Multivariate**	
	**HR (95% CI)**	***P***	**HR (95% CI)**	***P***
CHIC-HS risk stratification		0.005	2.07 (0.759–5.640)	0.026
Very low/low	1.000 (Reference)	0.004	NA	
Intermediate	1.856 (0.543–6.341)	0.023	NA	
High	5.437 (1.870–12.551)	0.011	NA	
PRETEST group	1.007 (1.335–4.841)	0.003	2.542 (1.335–4.841)	0.004
Histological subtype		<0.001		0.010
Fetal	1.000 (Reference)		1.000 (Reference)	
Embryonal	2.124 (0.027–0.576)	0.008	1.364 (1.075–1.731)	
Epithelial mixed	2.234 (0.046–0.698)	0.002	2.459 (0.370–16.344)	
Mixed epithelial/mesenchymal	4.492 (0.151–1.603)	0.019	3.354 (1.524–27.434)	
M (distant metastasis)	5.387 (2.131–13.620)	<0.01	0.150 (0.023–0.977)	0.047
F (multifocality)	4.527 (1.828–11.209)	0.001	NA	
P (PV involvement)	5.404 (2.167–13.474)	0.002	7.43 (2.134–25.911)	0.001
R (tumor rupture)	4.681 (1.071–10.465)	0.009	NA	
VPERF+	4.559 (1.726–12.040)	0.002	0.09 (0.010–0.967)	
Tumor vessel	2.503 (1.012–6.190)	0.040	NA	
Effusion	2.776 (1.112–6.927)	0.029	NA	
Radiomics signature	3.009 (1.963–4.613)	0.006	5.138 (1.268–16.495)	0.024

*HR, hazard ratio; CI, confidence interval; CHIC-HS, Children's Hepatic tumors International Collaboration-Hepatoblastoma systems; PV, portal vein; CHIC-HS, Children's Hepatic tumors International Collaboration-Hepatoblastoma Stratification; EFS, event-free survival*.

### Construction of a Rad-Score Based on a Radiomics Signature

**Figure 2:**
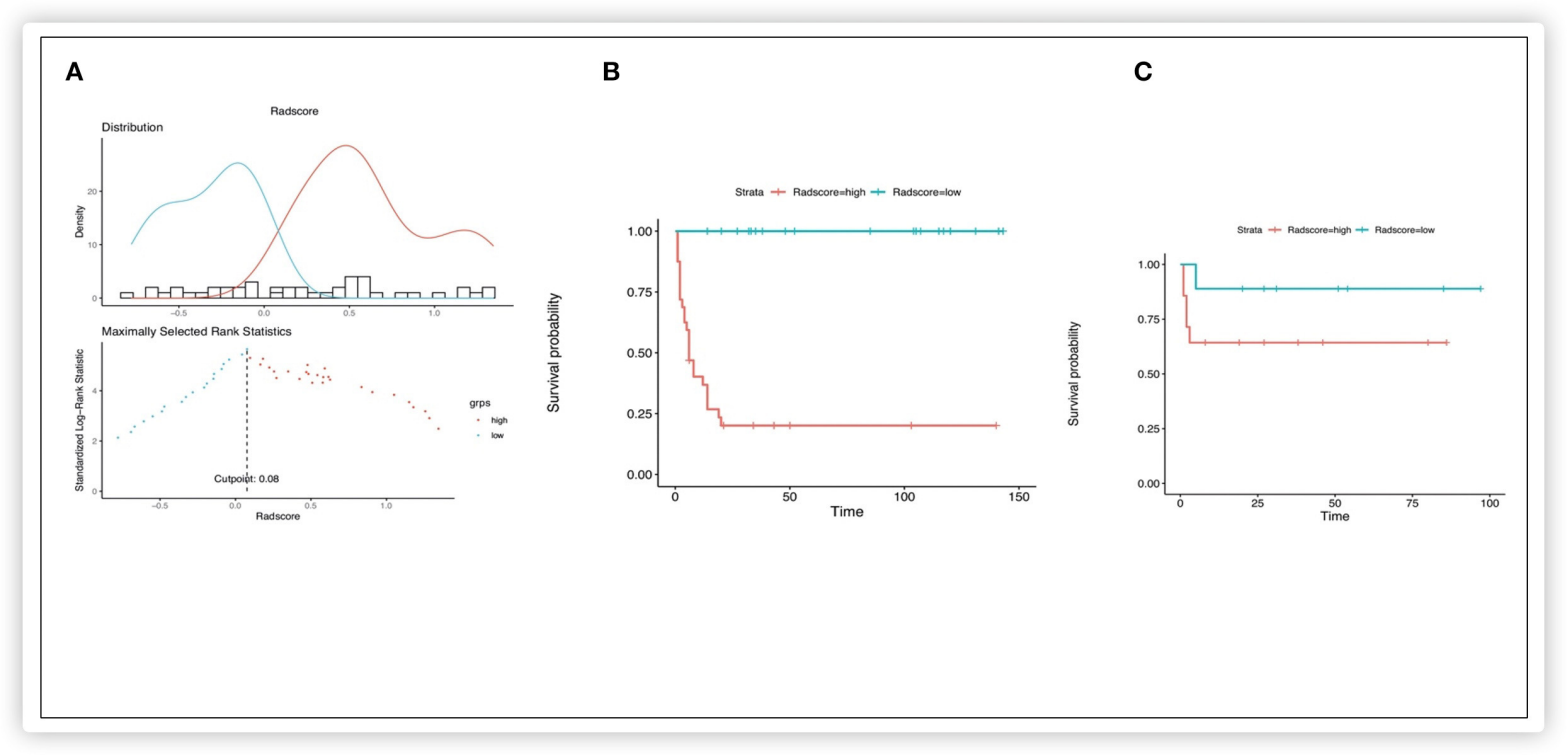
Kaplan-Meier survival analysis of event-free survival of patients with HB. Graphs show results of Kaplan-Meier survival analyses according to the radiomics score cutoff value **(A)** for patients in the training data set **(B)** and those in the validation data set **(C)**. A significant association of the radiomics score with the EFS was shown in the training data set, which was then confirmed in the validation data set.

Nine potential survival-related radiomics features from 92 significant features (*p* < 0.05) were documented. The nine features, namely, “TotalEnergy,” “LowGrayLevelZoneEmphasis,” “GrayLevelNonUniformity,” “LowGrayLevelZoneEmphasis,” “SmallAreaLowGrayLevelEmphasis,” “LowGrayLevelZoneEmphasis,” “ZoneEntropy,” “SizeZoneNonUniformityNormalized,” and “RunLengthNonUniformity,” were identified in the training cohort for the construction of a radiomics model and the calculation of Rad-score by the LASSO Cox regression analysis (Supplementary Table 1). The HR for the Rad-score was 3.009 (95% CI: 1.963–4.613) in the training cohort. According to the distribution of the Rad-score, we classified patients further into low-risk (Rad-score <0.08) and high-risk (Rad-score >0.08) groups using an optimal cutoff of 0.08 (Figure 2A). Survival analyses revealed a significant difference between these two risk-stratification groups (*P* < 0.001) in the training and validation cohorts (Figures 2B,C). Furthermore, survival analyses applied in all clinicopathologic subgroups (CHIC-HS, histology subtype, M, P, F, VPERF) demonstrated that these risk factors were significant for disease procession (*p* < 0.01) (Supplementary Figure 1).

### Performance of the Radiomics Nomogram Compared With the Clinical–Pathological Nomogram in Predicting EFS

**Figure 3:**
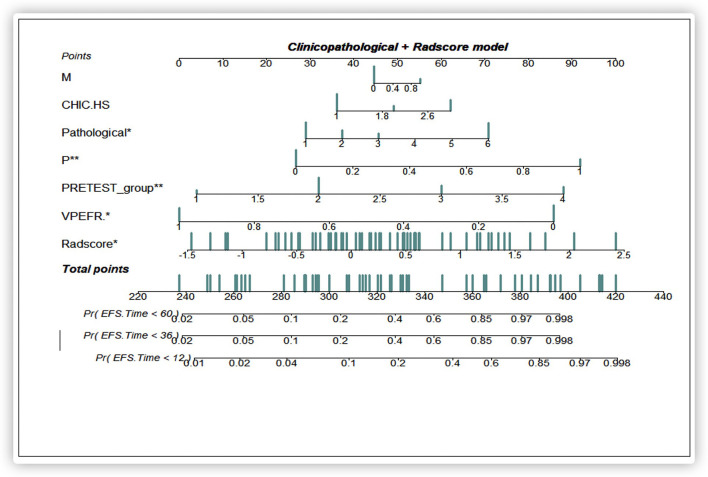
A prediction performance analysis with combined clinicopathologic-Rad-score of patients with HB. Significant codes: ^***^0; ^**^0.001; ^*^0.01; .0.05; ’ ’0.1; 1.

**Figure 4:**
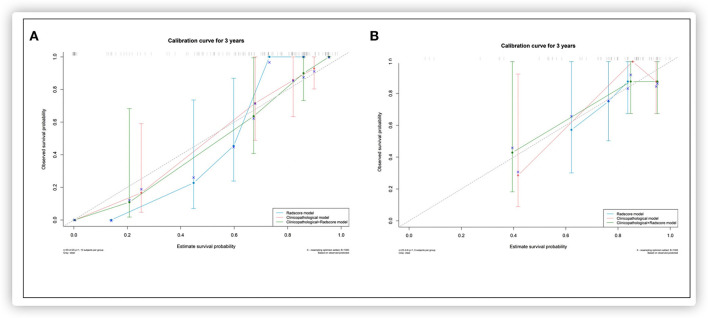
Calibration curves for each model in the internal validation **(A)** and external validation **(B)** set.

Based on the multivariate Cox regression analysis, three nomograms that incorporated the Rad-score and independent clinicopathologic factors and CR were generated (Figure 3). The radiomics nomogram exhibited good discrimination performance (C-Index: 0.810; 95% CI: 0.738–0.882) in the validation cohort, which was comparable with clinicopathologic factors (C-Index: 0.855; 95% CI: 0.790–0.920) without improvement in performance in EFS estimation. The combined model with Rad-score and clinicopathological factors achieved a significant incremental value (C-Index: 0.881; 95% CI: 0.829–0.933) for the accuracy of prognosis relative to the radiomics signature alone and the clinicopathologic factors alone. The estimates of the C-Index, of concordance probability, and of the Akaike information criterion (AIC) for the different prediction models are listed in Table 3. Moreover, the calibration curve indicated a satisfactory agreement between the outcome of survival prediction and actual survival at 3-year follow-up in the internal-validation cohort and external-validation cohort (Figure 4).

**Table 3:** Models performance.

**Model**	**C-index (95% CI)**	**Se (C-index)**	***P*-value**	**AIC**
Rad-score (1)	0.810 (0.738~0.882)	0.037	0.256 (1,2)	162.650
Clinicopathological (2)	0.855 (0.790~0.920)	0.033	0.211 (2,3)	154.446
Clinicopathological+Rad-score (3)	0.881 (0.829~0.933)	0.026	0.006 (1,3)	149.025

**Figure 5:**
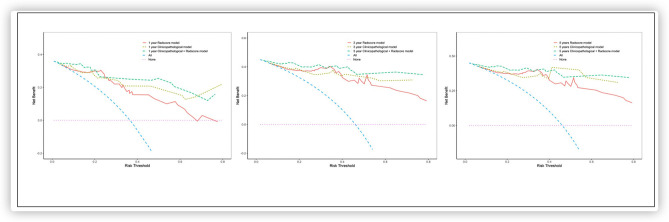
Decision curve analysis for each model between the observed 1-, 3-, and 5-year outcomes.

Analyses of decision curves showed that the combined model integrating the Rad-score and the clinicopathologic nomogram obtained higher clinical utility relative to that obtained using the radiomics signature alone or the clinicopathologic factors alone (Figure 5).

## Discussion

Patients with advanced HB carry a substantial risk of local relapse and distant metastasis even after resection. Indeed, only about one-thirds of patients with HB have a resectable tumor at initial presentation (2). Whether an optimal treatment regimen with adjuvant chemotherapy and surgery provides survival benefit is controversial and is largely dependent on the assessment of prognostic risk.

We developed a quantitative radiomics approach with CT for accurate pretreatment risk stratification in HB to assist in decision-making for clinical treatment. A LASSO Cox-based radiomics signature was demonstrated to be an independent risk factor for EFS in patients with HB. A radiomics model with specific radiomics features using pretreatment of CT-enhanced images showed similar performance to that of a postoperative clinicopathologic model for predicting EFS of HB. The combination of a nomogram with the Rad-score and clinicopathologic factors achieved better performance than that using the radiomics nomogram and the clinicopathologic nomogram for individualized EFS estimation. The result supports another possibility of preoperative evaluation before surgical pathology when Evans' surgical staging is used based on exploratory surgery for patients with HB (9). In addition, the patients were categorized into low- and high-risk groups by using the multiple feature-based Rad-score with significant differences in EFS, which would be an important supplement to the present PRETEXT and CHIC-HS systems for the evaluation of clinical risk factors. We investigated, for the first time, the use of radiomics features using quantitative CT images to predict disease progression in pediatric patients with HB.

In recent years, much attention has been paid to developing high-throughput screening to extract many quantitative features in medical imaging to assist the clinical diagnosis. Also, the radiomics signature has been shown to be validated robustly, be cost-effective, and be powerfully predictive of survival from solid tumors in several studies (18–22, 24, 25, 31–34). The selected radiomics signature provides a non-invasive, simple, and reproducible method for acquiring phenotypic information, which is not available using conventional imaging and which can be employed to predict survival outcomes. Reviews by scholars have suggested that intratumoral heterogeneity could be demonstrated by radiomics, which would imply a worse prognosis with relapse and metastasis (35, 36). A quantitative radiomics analysis by Aerts and colleagues revealed the radiomics signature and the interpretation of intratumor heterogeneity associated with the underlying gene expression (37). In the present study, the features of conventional CT images were extracted, and the analysis of dimensionality reduction was carried out by a LASSO regression algorithm to select the features that could best reflect the different components in HB. The LASSO approach was able to acquire all features with non-zero coefficients, which enhanced the interpretation and prediction accuracy of the model. The subset of the selected optimal features contained several first-order statistics features (which reflect the internal voxel intensity of lesions) and texture features (which reflect the gray-distribution characteristics in dimensional space and suggest the heterogeneity composition or distribution of lesions in the dimensional space). However, interpretation of the complex associations between radiomics features and biological processes in a tumor remains a challenge (38). Besides, considering the complex intervention, multiple interacting components, in the biological processes and the nature of malignancy, it is hard to correlate a single radiomics-based factor with a pathophysiological change in an intuitive manner. Hence, the multiple features based on the radiomics signature show significantly greater estimation outcome than that of any selected feature alone. In the present study, nine features of a radiomics signature in 88 patients achieved a favorable performance for the assessment of survival from HB. In addition, the multiple-component radiomics signature from the training cohort was externally validated in the test cohort using different device brands and at different institutions, thereby illustrating the repeatability and stability of this model.

In recent decades, an increasing number of studies focusing on the association between several risk factors and HB survival have been published (9–12). Although prognostic factors and outcomes were stated, the studies failed to provide a new prognostic model (2). The CHIC group developed unified risk stratification for patients with HB. Several factors, namely AFP, PRETEXT group, and PRETEXT annotation factors, were shown to affect patient survival (4). However, the present CHIC-HS system may be awkward to use in clinical practice because there are many variables in its complex stratification system. Also, the reasons for some factors eliciting a worse outcome (e.g., older age) are not entirely clear. In addition, the prognostic importance of pathological subtypes has not been included in study groups. Some histology types have been associated with the prognosis in clinical trials (9, 39). Hence, comparisons between any outcome and prognostic factors between groups are challenging and unreliable (13). In accordance with the CHIC-HS, we defined identical risk factors in the present study as well as histology types. Although histology subtypes were incomplete (small-cell undifferentiated and macrotrabecular types were absent), the results are consistent with data from studies that reported a better prognosis with a pure fetal well-differentiated type and a worse prognosis with a non-pure fetal type (40). We also investigated the performance of morphologic characteristics using conventional imaging as a preoperative prognostic factor for potential association with outcome of HB, as described in an earlier study (17, 41). However, none of the morphologic features on imaging were included in the regression models in the present study; their prognostic value and the association with EFS should be validated in additional studies. We incorporated CHIC-HS-related risk factors and morphologic features into a clinicopathologic model. We found that all variables remained as predictors to identify an increased risk, except the PRETEXT annotation factor E; these observations are in accordance with the results of previous studies (4, 13). The multivariate Cox regression analysis indicated that the PRETEXT annotation factor, P, was the strongest negative independent prognostic factor and that factor M showed borderline significance in our cohort; these data are consistent with results from other similar studies (27).

Nomograms have been developed for some cancers and are accepted as reliable tools for the prediction of risk for individuals (42, 43). Based on the abovementioned risk factors, we incorporated the radiomics signature and clinicopathologic characteristics to develop and validate a radiomics–clinicopathologic nomogram to improve the accuracy of prognosis prediction for patients with HB. Overall, the results from the combined nomogram displayed more robust power for predicting EFS in HB than that of the radiomics model alone or the clinicopathologic model alone, with a higher C-index, better calibration, and improvement in the net reclassification. Furthermore, the decision curve analysis indicated the benefit of using combined model beyond the clinical staging system across the appropriate range of reasonable threshold probabilities. This result suggested that the radiomics signature reinforced the prognostic ability of the staging system, thereby adding prognostic value to clinicopathologic risk factors. The concept of this combination model has been described previously (34, 44). However, the combination model mentioned earlier was employed to facilitate personalized treatment for patients with HB and provide pediatricians with a powerful tool for making clinical decisions. The radiomics nomogram (which contained the Rad-score) yielded a C-Index of 0.810 (95% CI: 0.738–0.882) for EFS prediction in the validation cohort, and this C-Index was similar to that of the clinicopathologic model. This observation suggested that the Rad-score might contain clinical information due to the extraction of high-dimensional features that can capture prognostic information. Thus, the Rad-score could be used as a surrogate biomarker to improve the prognostic ability before treatment. In addition, the Rad-score could be used to stratify patients into low- and high-risk groups. Patients with higher Rad-scores had worse EFS, which suggested that low-risk patients would receive radical hysterectomy. For the high-risk group, intensive treatment could elicit greater survival benefits. Thus, the Rad-score may be an efficient tool to enable personalized treatment before surgery.

The present study had five main limitations. First, the sample size was small (particularly the external-validation cohort). Second, the study was retrospective. Third, missing information and a small number of clinicopathologic factors [e.g., age group (>8 years) or histology subtypes] may have led to a performance bias. Fourth, the underlying connection between the radiomics signature and biological information was not investigated. Finally, delineation of RoIs was processed using a manual method. The exploitation of auto-segmentation could be an efficient and accurate method for lesion identification in future radiomics research.

In conclusion, a radiomics signature can be used as a biomarker for risk stratification in patients with HB. A nomogram integrating the radiomics signature, the staging system, and other clinicopathologic risk factors for EFS estimation may be a valuable tool for guiding individual pretreatment for patients with HB.

## Data Availability Statement

The raw data supporting the conclusions of this article will be made available by the authors, without undue reservation.

## Ethics Statement

The studies involving human participants were reviewed and approved by Institution Ethics Committee of China West second hospital of Sichuan University. Written informed consent to participate in this study was provided by the participants' legal guardian/next of kin. Written informed consent was obtained from the individual(s), and minor(s)' legal guardian/next of kin, for the publication of any potentially identifiable images or data included in this article.

## Author Contributions

YJ, YX, and YG: conceptualization. JS: data curation. JS and YC: formal analysis. YC: investigation. LX and XG: methodology. YG: project administration and YG: supervision. YJ: writing—original draft. YJ and YG: writing—review and editing. All authors: contributed to the article and approved the submitted version.

## Conflict of Interest

The authors declare that the research was conducted in the absence of any commercial or financial relationships that could be construed as a potential conflict of interest.
