# Supplementary material for: Preoperative Assessment for Event-Free Survival With Hepatoblastoma in Pediatric Patients by Developing a CT-Based Radiomics Model
Source: Front Oncol. 2021 Apr 16;11:644994. doi: 10.3389/fonc.2021.644994 (PMC8086552; doi:10.3389/fonc.2021.644994)
Supplement: Supplementary file 1 [file Table_1.docx]

**Supplementary Table 1** **|** List of selected radiomics features derived.

| Feature Category | Contrast-enhanced CT | Correlation Coefficient |
| --- | --- | --- |
| First Order Statistic | Gradient_firstorder_Total Energy | 0.050 |
| Wavelet | LHH_gldm_LargeDependenceLowGrayLevelEmphasis | 0.116 |
|  | LHH_glszm_GrayLevelNonUniformity | 0.376 |
|  | HLL_glszm_LowGrayLevelZoneEmphasis | 0.407 |
|  | HLL_glszm_SmallAreaLowGrayLevelEmphasis | 0.141 |
|  | LLH_glszm_LowGrayLevelZoneEmphasis | 0.062 |
|  | HLH_glszm_ZoneEntropy | 0.186 |
|  | HHL_glszm_SizeZoneNonUniformityNormalized | 0.179 |
|  | LLL_glrlm_RunLengthNonUniformity | 0.336 |

*GLSZM, Gray Level Size Zone Matrix; GLDM, Gray Level Dependence Matrix; GLRLM, Gray Level Run Length Matrix; LHH, HLL, LLH, HLH, HHL and LLL are subtypes of wavelet filters.*
