# Supplementary material for: Preoperative Assessment for Event-Free Survival With Hepatoblastoma in Pediatric Patients by Developing a CT-Based Radiomics Model
Source: Front Oncol. 2021 Apr 16;11:644994. doi: 10.3389/fonc.2021.644994 (PMC8086552; doi:10.3389/fonc.2021.644994)
Supplement: Supplementary file 2 [file Data_Sheet_1.docx]

**SUPPLEMENTARY MATERIAL**

**Supplementary Material I. CT Image Acquisition Parameters**

All patients performed contrast-enhanced abdomen CT by using different multidetector row CT (MDCT) systems (NeuViz 128, Neusoft, CHINA; Discovery CT750 HD, GE Healthcare, USA). The scanning parameters were described as follow: 180-200 mAs; 120 kV; 0.5- or 0.6-second rotation time; slice thickness and interval for axial images, 5 mm/5 mm; 8×2.5 mm or 64×0.625 mm detector collimation; field of view (FOV): 350×350, 500×500 mm. After routine nonenhanced CT, arterial and portal venous-phase contrast-enhanced CT were performed after delays of 25 s and 62s after the arterial phase scanning. Portal venous phase (PV) Digital Imaging and Communications in Medicine (DICOM) images (thickness: 2.5-5mm) were acquired from the picture archiving and communication system (PACS) without any compression for image feature selection for the reason of well differentiation of the lesion from the adjacent normal liver parenchyma.

**Supplementary Material II. CT Image normalization Processing Step**

Imaging preprocessing to obtain more robust radiomics features consists of two steps: Step 1.We used the following formula to normalize the intensity of the image to minimize the change in CT intensity collected by machines with different parameters(i is the original intensity; *F*(i) shows the normalized intensity; *µ*_𝒾_ is the mean value of the image intensity values; σ_i_ indicates the standard deviation of the image intensity values; *s* is an optional zoom and is set to 1 by default). Normalization is for the whole image, not just the region of segmentation.

$$F(i)=\frac{s(i-\mu_{i})}{\sigma_{i}}$$

Step 2. In order to eliminate the intrinsic dependence of radiomics features on voxel size, the resampling method with a linear interpolation algorithm was used to normalize voxel size.

**Supplementary Material III. Extracted Radiomics Features**

An open source software “pyradiomics” package in Python (version 3.8.1, <https://pyradiomics>. readthedocs.io/) was employed to extract a total of 1409 quantitative imaging features from CT PV images, which grouped into four groups. (1) First-order statistics (n = 18), described the intensity information in the CT image region of interest, such as mean, standard deviation, variance, maximum, median, range, etc. (2) Shape features (n = 14), which reflected the shape and size of the region, such as volume, surface area, compactness, aximum diameter, etc. (3) Texture features, which could quantify regional heterogeneity differences, such as gray-level co-occurrence matrix (GLCM, n = 24), gray-level size zone matrix (GLSZM, n = 16), gray-level dependence matrix (GLDM, n = 14), neighborhood gray-level dependence matrix (NGLDM, n=5), and gray-level run-length matrix (GLRLM, n = 16). (4) higher-order statistical features, contained 1302 features, including the first-order statistics and texture features derived from wavelet transformation of the original images: logarithm, exponential, gradient, square, square root, local binary patterns (LBP), the wavelet transform decomposes the tumor area image into low-frequency components (L) or high-frequency components (H) in the three directions of the x, y, and z axes. Eight types of wavelet features were obtained and labeled as LLL, LLH, LHL, LHH, HLL, HLH, HHL, HHH according to their different decomposition orders.
