# Supplementary figures and images for: Preoperative Assessment for Event-Free Survival With Hepatoblastoma in Pediatric Patients by Developing a CT-Based Radiomics Model
Source: Front Oncol. 2021 Apr 16;11:644994. doi: 10.3389/fonc.2021.644994 (PMC8086552; doi:10.3389/fonc.2021.644994)

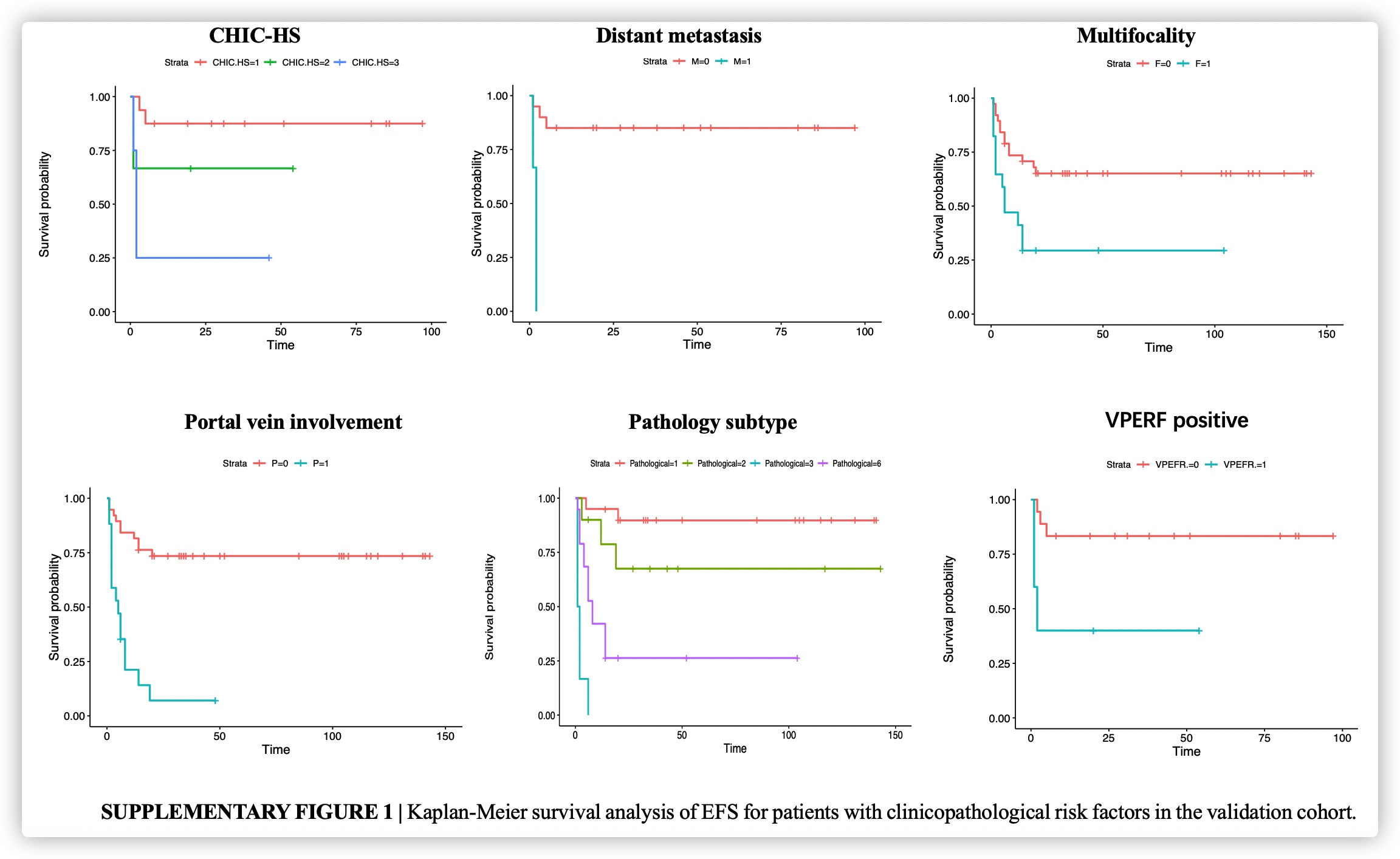

Supplement: Supplementary file 3 [file Image_1.jpg]
